# Inhibitory Effects of *Laminaria japonica* Fucoidans Against Noroviruses

**DOI:** 10.3390/v12090997

**Published:** 2020-09-07

**Authors:** Hyojin Kim, Chae Yeon Lim, Dan Bi Lee, Jong Hyeon Seok, Kyung Hyun Kim, Mi Sook Chung

**Affiliations:** 1 Department of Food and Nutrition, Duksung Women’s University, Seoul 01369, Korea; hyojinkim912@duksung.ac.kr (H.K.); soang205@duksung.ac.kr (C.Y.L.); 2 Department of Biotechnology and Bioinformatics, Korea University, Sejong 30019, Korea; ann21010@korea.ac.kr (D.B.L.); tjrwhdgus@korea.ac.kr (J.H.S.)

**Keywords:** norovirus, P domain, antiviral, *Laminaria japonica*, fucoidan

## Abstract

Norovirus is the leading cause of nonbacterial foodborne disease outbreaks. Human noroviruses (HuNoVs) bind to histo-blood group antigens as the host receptor for infection. In this study, the inhibitory effects of fucoidans from brown algae, *Laminaria japonica* (LJ), *Undaria pinnatifida* and *Undaria pinnatifida* sporophyll, were evaluated against murine norovirus (MNoV), feline calicivirus (FCV) and HuNoV. Pretreatment of MNoV or FCV with the fucoidans at 1 mg/mL showed high antiviral activities, with 1.1 average log reductions of viral titers in plaque assays. They also showed significant inhibition on the binding of the P domains of HuNoV GII.4 and GII.17 to A- or O-type saliva and the LJ fucoidan was the most effective, reaching 54–72% inhibition at 1 mg/mL. In STAT1^−/−^ mice infected with MNoV, oral administration of the LJ fucoidan, composed of mainly sulfated fucose and minor amounts of glucose and galactose, improved the survival rates of mice and significantly reduced the viral titers in their feces. Overall, these results provide the LJ fucoidan can be used to reduce NoV outbreaks.

## 1. Introduction

Human noroviruses (HuNoVs) are a leading cause of infectious nonbacterial foodborne diseases [1,2]. Globally, it affects all age groups and causes severe sufferings in the elderly and groups under five [1,3]. The transmission of HuNoVs occurs mainly by eating contaminated foods or by contact with contaminated hands. A low dose of 18–1000 HuNoV particles can infect humans [4]. NoVs were recently reclassified into 10 genogroups (GI–GX) and 49 genotypes [5]. Among them, the GII.4 and GII.17 genotypes have been the predominant cause of foodborne outbreaks for the past two decades and spread worldwide since 2014, respectively [6].

NoV, a non-enveloped virus, belongs to the *Caliciviridae* family. The positive-stranded viral RNA genome is organized into three open reading frames (ORFs). ORF1 and ORF2 encode a polyprotein and the major structural protein (VP1), respectively and ORF3 encodes a minor structural protein (VP2), forming a portal-like assembly [7,8]. VP1 has shell (S) and protruding (P) domains of which the latter is divided into P1 and P2 subdomains; the P2 subdomain contains the receptor binding site and determinants of antigenicity [8,9].

HuNoV can be cultured in human intestinal enteroids [10,11]. However, the accessibility of the cultivation method is limited by the high cost and labor-intensive nature. In this context, murine NoV (MNoV) and feline calicivirus (FCV) have been used as surrogates for HuNoV in numerous studies [12,13,14]. HuNoVs recognize histo-blood group antigen (HBGA) as the host receptor for infection [15,16]. The HBGA is a fucose-containing glycan attached to proteins and lipids on epithelial cells, where they serve as receptors and also as receptor analogs in saliva and human milk oligosaccharides [17,18,19]. It was reported that 2′-fucosyllactose and 3-fucosyllactose in human milk bind to the HBGA-interaction sites on the P domain of HuNoVs [20,21,22,23].

Brown algae, *Laminaria japonica* (LJ)—also known as *Saccharina japonica*, *Undaria pinnatifida* (UP) *and Undaria pinnatifida* sporophyll (UPS)—contain fucoidans and phlorotannins [24,25]. Fucoidan is a fucose-containing sulfated polysaccharide [26]. It has numerous proven bioactivities—antioxidant [27], anticoagulant [28], antiviral [29,30], anticancer [31] and antibacterial [26] activities. Nevertheless, the antiviral effects of fucoidans from brown algae against HuNoV and MNoV and FCV have not been reported. Here, we report significant inhibitory activities of the fucoidans from LJ, UP and UPS by blocking the HuNoV P domain binding and reducing viral titers. In addition, STAT1^−/−^ mice infected with MNoV showed improved survival rates and reduced viral titers in the feces by the oral administration of the LJ fucoidan.

## 2. Materials and Methods

### 2.1. Cells and Viruses

MNoV-1.CW1 (MNoV), FCV-F9 (FCV), RAW 264.7 (RAW) cells and Crandell Reese feline kidney (CRFK) cells were prepared as described previously [32]. RAW and CRFK cells were maintained in Dulbecco’s modified Eagle’s medium (DMEM) (Gibco BRL, Karlsruhe, Germany) with fetal bovine serum (FBS) (Sigma-Aldrich, St. Louis, MO, USA) in a CO_2_ incubator at 37 °C. The viruses were grown in RAW or CRFK cells.

### 2.2. Extraction of Fucoidans from Brown Algae

The fucoidans of LJ, UP and UPS were extracted as described previously [33]. The alga was washed with distilled water, air-dried and crushed into particles of 20-mesh size, which was extracted in 50 mM HCl for 2 h. The extract was centrifuged at 11,000× *g* for 30 min and the supernatant was neutralized with NaOH, to which 1% CaCl_2_ was added and centrifuged at 11,000× *g* for 20 min. For fucoidan precipitation, two volumes of 95% ethanol were added to the supernatant, followed by centrifuged again at 11,000× *g* for 20 min. The precipitated fucoidan was lyophilized and the yield (%) was calculated by the weight after lyophilization divided by the weight of the algae sample and multiplied by 100. For antiviral activity analysis, the lyophilized fucoidan was diluted with sterile water and passed through a 0.20-µm filter.

### 2.3. Cytotoxicity

Cytotoxicity of the fucoidan was determined as described previously [32]. Briefly, the prepared fucoidan was added to 90% confluent monolayer of CRFK or RAW cells in 96-well plates and incubated in a CO_2_ incubator at 37 °C for 24 h. The 3-(4,5-dimethylthiazol-2-yl)-2,5-diphenyltetrazolium bromide (MTT, Sigma-Aldrich) solution was added to each well and incubated, which was followed by addition of dimethyl sulfoxide (Sigma-Aldrich). Absorbance at 570 nm was measured using a microplate reader (SpectraMax M2, Molecular Devices, San Jose, CA, USA). The experiments were performed in triplicate.

### 2.4. Plaque Assay

The effect of fucoidan on viral titer reductions was analyzed by plaque assays. The pretreatment of MNoV or FCV was conducted by incubating the virus suspension (6−7 log plaque-forming unit (PFU)/mL) with an equal volume of the fucoidan at room temperature for 3 h. The incubated suspension was diluted in DMEM and inoculated onto RAW or CRFK cells at 37 °C for 3 h. After inoculation, inocula were discarded, followed by the incubation of DMEM containing agarose and FBS for 24–48 h and 4% formaldehyde was added to the cell monolayer. Finally, 0.5% crystal violet was added and the number of plaques was counted. DMEM and a commercial fucoidan from U. pinnatifida (≥95% purity, Sigma-Aldrich) were served as untreated and positive controls, respectively. The experiments were performed in triplicate.

### 2.5. Expression and Purification of HuNoV P Domains

The DNA fragments encoding HuNoV GII.4 (Hu/GII.4/Hiroshima/55/2005/JPN) and GII.17 (Hu/GII/JP/2015/GII.P17_GII.17/Kawasaki308) P domains (GenBank accession number BAI49908.1 and LC037415.1, respectively) were synthesized by Macrogen (Seoul, Korea). P domains of HuNoV GII.4 and 17 were expressed and purified described as previously with minor modifications [34]. Each gene was cloned into the NdeI and BamHI sites of the pET14b vector (Novagen, Madison, WI, USA) and transformed into Escherichia coli BL21 (DE3) (Novagen). The cells were collected by centrifugation at 3500× *g* for 15 min, sonicated and centrifuged at 12,000× *g* for 20 min. The supernatant containing the P domain with an N-terminal His-tag was purified by affinity chromatography and size exclusion chromatography using nickel-nitrilotriacetic acid (Qiagen, Hilden, Germany) and Superdex 200 10/300 (GE HealthCare, Uppsala, Sweden) columns, respectively (Figure S1). The molecular masses of the P domains from HuNoV GII.4 and GII.17 were 35 kDa.

### 2.6. Enzyme-Linked Immunosorbent Assay (ELISA)

The inhibitory effects of the fucoidans on the binding of HuNoV GII.4 and GII.17 P domains to saliva were analyzed as reported by Weichert et al. [23] with minor adjustments. Two types of saliva samples (A- and O-type) were obtained from volunteers at Duksung Women’s University. The supernatant (100 µL) of saliva was coated in Maxisorp plates at a dilution of 1:100 in PBS. After blocking with 5% non-fat dried milk overnight at 4 °C, serially diluted P domains were added and incubated for 2 h at room temperature. The P domain was incubated with the fucoidan prior to binding to the saliva at 4 °C overnight. The biotin-conjugated anti-NoV antibodies against GII.4 or GII.17 P domain (R-Biopharm AG, Darmstadt, Germany) were used at a dilution of 1:20,000 in PBS with non-fat dried milk, followed by the addition of streptavidin poly-peroxidase conjugated antibody (R-Biopharm AG) diluted in PBS-non-fat dried milk at a dilution of 1:40,000. o-Phenylenediamine and H_2_O_2_ were added and incubated for 30 min at room temperature, which was stopped by adding 3 N hydrochloric acid and absorbance was read at 450 nm with a microplate reader (SpectraMax M2). The P domains derived from HuNoV GII.4 and GII.17 were able to bind to A- and O-type saliva in a concentration-dependent manner at 0.19−12 µg/mL and thereby the fucoidan inhibition was examined using 3−6 µg/mL P domains, giving optimum absorbances (Figure S2). The absorbance at 450 nm of the untreated P domain was taken as 100% binding to the saliva and the inhibition (%) was calculated: [1 − (Absorbance_450_ of treated P domain/Absorbance_450_ of untreated P domain)] × 100. The experiments were performed in triplicate.

### 2.7. Carbohydrate Composition of the LJ Fucoidan

Trifluoroacetic acid (TFA, Sigma-Aldrich) was used to hydrolyze LJ fucoidan as described previously [35]. The LJ fucoidan and 2 M TFA were incubated in screw-cap vials, kept at 121 °C for 2 h and dried under a nitrogen stream. The dried samples were diluted with distilled water and passed through a 0.20-µm filter, which was used for high-performance liquid chromatography (HPLC) analysis.

Dionex ICS-5000 ion chromatography (Thermo Scientific Dionex; Waltham, MA, USA) with a CarboPac SA 10 column (4 × 250 mm, Dionex; Sunnyvale, CA, USA) was used to quantify the monosaccharide of the LJ fucoidan. 200 mM NaOH was used as the mobile phase and the flow rate was 0.5 mL/min. Standard monosaccharides such as L-fucose, D-galactose, D-glucose, D-mannose, L-rhamnose monohydrate and D-xylose were obtained from Sigma-Aldrich.

### 2.8. Sulfate Contents of the LJ Fucoidan

The sulfate contents of the LJ fucoidan were measured as described previously [36]. The LJ fucoidan was hydrolyzed with 10 mg of 2 M TFA in a screw-cap vial and kept for 2 h at 121 °C. The barium-gelatin solution was prepared by adding 0.75 g of gelatin (Sigma-Aldrich) in 250 mL of boiling water, cooling and mixing with 10 g of barium chloride (BaCl_2_·2H_2_O, Sigma-Aldrich). The barium-gelatin solution and 0.5 N of hydrochloric acid (Sigma-Aldrich) were mixed in a 1:1 ratio. The mixture (50 µL) and hydrolyzed sample (250 µL) were added into a microtiter plate and determined the absorbance at 450 nm. The standard curve was generated using a serial dilution of anhydrous sodium sulfate (Na_2_SO_4_). The experiments were performed in triplicate.

### 2.9. In Vivo Mouse Experiment

All animal experiments were performed in accordance with the recommendations in the Guide for the Care and Use of Laboratory Animals from the Animal, Plant and Fisheries Quarantine and Inspection Agency, Republic of Korea. The study protocol was approved by the Institutional Animal Care and Use Committee of Duksung Women’s University (2019-003-003). All efforts were made to reduce the suffering of the animals and sacrificed upon a body weight loss of 20% at the utmost. B6.129S(Cg)-Stat1^tm1Dlv^/J (referred to as STAT1^−/−^ hereafter) mice from the Jackson Lab (Bar Harbor, ME, USA) were bred and housed at the animal lab under specific-pathogen-free conditions. Female STAT1^−/−^ knockout mice at 5–8 weeks of age were randomly distributed per group and inoculated perorally with 3 × 10^4^ PFU of MNoV. Treatment with the fucoidan was initiated immediately after infection (n = 4) with a dose of 40 mg/kg/day until 4 days post-infection (dpi) by oral gavage. PBS was administered to untreated control mice (n = 4). Treated and untreated mice were kept separate in independently ventilated cages for all the experiments. Mice were weighed daily and stools were collected during experimental period. For fecal MNoV titers, homogenates of the samples were centrifuged at 4000× *g* and 4 °C for 5 min. The supernatant was collected to be the fecal suspension. RAW cell monolayers were infected at 37 °C for 2 h with 500 μL of the fecal suspension in 1:10 dilution in a 24-well plate. After infection, cells were washed and overlaid with DMEM containing 1% agarose for 48 h at 37 °C in a 5% CO_2_ incubator. Plaques were counted after 0.5% crystal violet staining.

### 2.10. Statistical Analysis

Statistical analyses were performed using IBM SPSS Statistics (IBM Corp, New York, NY, USA). Data were expressed as mean±SD. Statistical analysis was performed with a t-test. Significance level was indicated by * *p* < 0.05 and ** *p* < 0.01. For multiple comparisons, the data were analyzed by ANOVA and the mean values were compared with Tukey’s test at the 5% significance level. The experiments in this study were conducted in triplicate.

## 3. Results

### 3.1. Preparation of the Fucoidans and Their Effects on Cell Viability

The fucoidans of LJ, UP and UPS were obtained by grinding each alga, followed by acid treatment and neutralization. They were then treated with CaCl_2_ to remove alginic acid, precipitated by ethanol and lyophilized. The recovery yields were 1.8%, 0.6% and 5.7%, respectively. RAW and CRFK cell viabilities were above 90% after 24 h incubation at 1 mg/mL of these fucoidans (Figure S3).

### 3.2. In Vitro MNoV and FCV Reduction by the Fucoidans

The LJ, UP or UPS fucoidan showed 0.2−0.4 log reduction of MNoV at 100 µg/mL and 0.7−1.4 log reduction at 1000 µg/mL (Table 1). A commercial fucoidan (≥95% purity), used as a positive control, caused 0.4−1.1 log reduction at the same concentration. For FCV, the fucoidans of LJ, UP and UPS showed 0.2−0.5 log reductions at 100 µg/mL and 0.8−1.3 log reductions at 1000 µg/mL. The control fucoidan also showed inhibitory activities against FCV, similar to those against MNoV. The fucoidans of LJ and UPS thus showed higher antiviral effects than that of the UP fucoidan against MNoV or FCV.

### 3.3. Inhibitory Effects of the Fucoidans on Binding of HuNoV P Domains to Receptors

**Figure 1:**
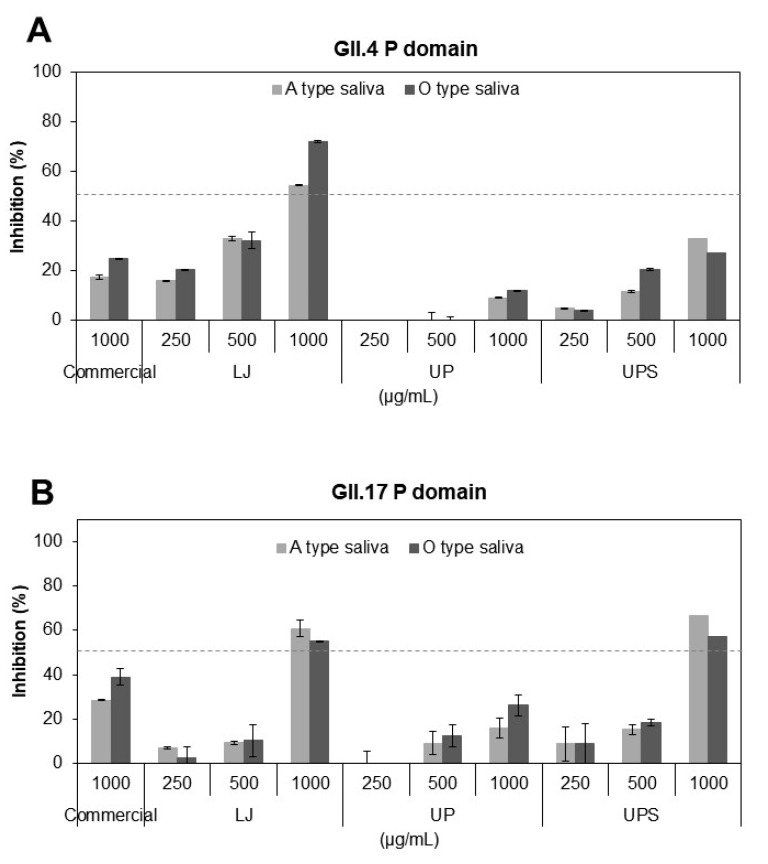
Inhibition of binding of the P domains of human norovirus (HuNoV) GII.4 (**A**) or GII.17 (**B**) to A- or O-type saliva by the fucoidans of *Laminaria japonica* (LJ), *Undaria pinnatifida* (UP) *and Undaria pinnatifida* sporophyll (UPS). Inhibition was quantified by ELISA using anti-NoV antibodies against GII.4 and GII.17 P domain. A commercial fucoidan (≥95% purity) was used as a positive control. The dashed line indicates 50% inhibition.

The binding of the P domains derived from HuNoV GII.4 and GII.17 to A- or O-type saliva was inhibited by the fucoidans in a concentration-dependent manner at 250–1000 µg/mL (Figure 1A,B). The LJ fucoidan at 1000 µg/mL showed 54–72% inhibition against the GII.4 P domain binding to saliva, higher than those by the UPS and UP fucoidans, whereas the GII.17 P domain binding was inhibited by the LJ and UPS fucoidans by 55–66%, more significantly than the UP fucoidan. The positive control, a commercial fucoidan at the same concentration showed 18–39% inhibition against the P domain binding to saliva. The LJ fucoidan was thus shown to inhibit the attachment of the P domains to receptors most effectively among the fucoidans used in this study.

### 3.4. The Chemical Composition of the LJ Fucoidan

The monosaccharide composition and sulfate content of the LJ fucoidan were analyzed by LC and the barium-gelatin method, respectively. On a dry matter basis, 23.6% fucose was identified in the LJ fucoidan, as a dominant monosaccharide and galactose, mannose, glucose and xylose were minor monosaccharides reaching 0.3–2.8% content (Table 2). The sulfate content in the LJ fucoidan was 25.8%, strongly suggesting that the LJ fucoidan is a fucose-containing sulfated polysaccharide.

### 3.5. Improvement of Survival Rates in Mice by the LJ Fucoidan

**Figure 2:**
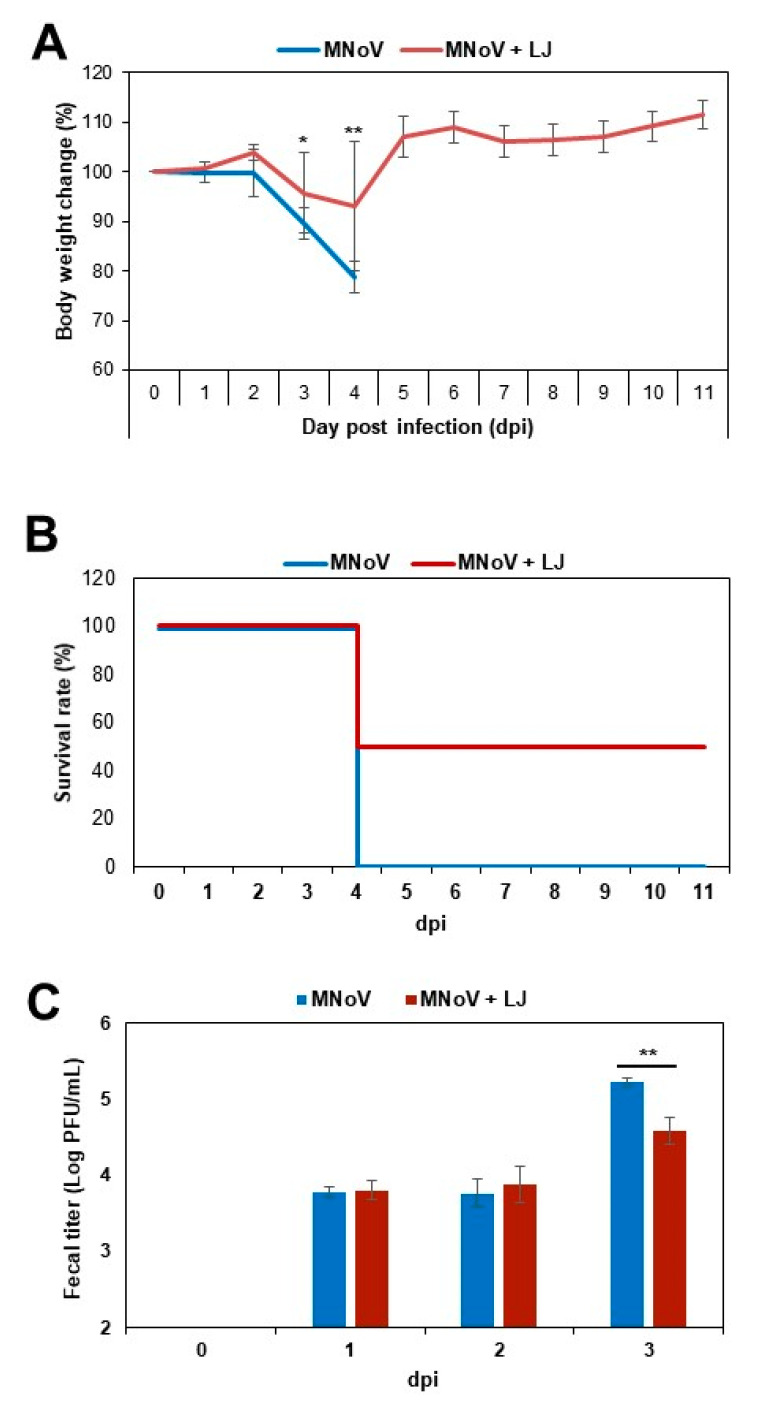
In vivo efficacy of fucoidans of *Laminaria japonica* (LJ). (**A**) Body weight changes, (**B**) survival rate until 11 dpi and (**C**) fecal viral titers. STAT1^−/−^ mice were inoculated perorally with 3 × 10^4^ PFU of MNoV and a dose of 40 mg fucoidan/kg/day (n = 4 per group) was administered by oral gavage immediately after infection until 4 days post-infection (dpi). A PBS group was used as control. Asterisk indicates significant difference (* *p* < 0.05; ** *p* < 0.01).

The STAT1^−/−^ mice were infected perorally with 3 × 10^4^ PFU of MNoV and the LJ fucoidan was administered immediately after infection until 4 dpi. The mice in the MNoV control group started to show a significant body weight loss with marked anorexia and behaviors consistent with physical discomfort or lethargy such as huddling, hunching and fur ruffling at 3 dpi. At 4 dpi, little fecal samples were found in the MNoV-infected mice and the weight loss reached 21% at 4 dpi, when they were humanely euthanized. In contrast, there was little weight loss in the fucoidan-treated mice at 3 dpi and rapidly regained body weight at 5 dpi (Figure 2A). The survival rates were 50% in the fucoidan-treated mice (Figure 2B).

When the viral titers in the feces were determined by plaque assay using RAW cells, MNoV was detected in the feces in the control and fucoidan-treated groups at 1 dpi. However, the fucoidan-treated mice showed more reduced titers (a 0.6 log reduction) of MNoV at 3 dpi, compared to those in the control group (significant differences by t-test, *p* < 0.01) (Figure 2C).

## 4. Discussion

HuNoV has emerged as the predominant cause of non-bacterial foodborne diseases, since the introduction of rotavirus vaccines. The annual global healthcare costs of HuNoV infections are estimated to be $4.2 billion [37]. NoV outbreaks are primarily reported in institutional meal settings such as in schools, child care centers and healthcare facilities [1]. Although there is no effective antivirals or vaccines commercially available for NoV, promising results have been reported with antiviral food materials and their components such as blueberry juice [38], green tea [39], grape seed extract [40], black raspberry seed and kimchi ingredients [32], curcumin [41] and resveratrol [42].

In this study, plaque assays demonstrated that the fucoidans from brown algae, LJ, UP and UPS, reduced the MNoV and FCV titers in a pretreatment mode. The LJ, UP and UPS fucoidans may have antiviral effects against MNoV and FCV by interfering with virus attachment to host cell receptors. The fucoidans may also inhibit the binding to HBGAs of the P domains derived from HuNoV GII4 and GII.17, by showing that they efficiently blocked the P domain binding to saliva samples of A- and O-type in a concentration-dependent manner. In particular, the LJ fucoidan at 1000 µg/mL exhibited strong inhibitory effects, reaching 54–72% inhibition against the binding of the P domains to saliva. These observations were in accordance with the inhibition of NoV binding in the previous report [43], where the fucoidan derived from *Fucus vesiculosus* functions as a structural decoy for HuNoV binding. Human milk oligosaccharides have been suggested as promising antivirals, which mimic HBGA [9,21]. Citrate was also reported to interact with the HBGA binding site of the NoV P domain [44]. Further studies are necessary to elucidate the inhibition mechanism of the fucoidan against MNoV or FCV.

To date, there has been a limit in animal models for HuNoV. While MNoV does not cause serious clinical disease in wild-type mice, it causes fatal disease in mice lacking interferon (IFN)-α, β and γ receptors or one of the key transcription factors in IFN signaling pathways, the signal transducer and activator of transcription 1 (STAT1) molecule (STAT1^−/−^ mice) [45]. Oral infection of STAT1^−/−^ mice with MNoV showed rapid virus replication and severe weight loss and, in addition, high levels of viral RNA or PFU in the proximal small intestine and feces by 3 dpi [45,46]. A significant proportion of infected animals ultimately succumbed to the disease. It was also reported that the treatment with 2′-C-methylcytidine (2CMC) 1 h before infection exhibited protection from MNoV-induced diarrhea and mortality in AG129 mice (129/Sv mice deficient in IFN-α, β and γ receptors) [47]. The 2CMC treatment of AG129 mice resulted in a 2 log reduction of viral RNA copies shedding in stool at 3 dpi. In the present mouse examination, the mice in the control group showed symptoms of NoV illness such as weight loss, hunched posture and ruffled fur at 3 dpi, which was consistent with the results of the AG129 mice [47]. The illness progressed rapidly and all the mice of the control group were euthanized at 4 dpi. The LJ fucoidan-treated mice exhibited a significant reduction of virus titers in the feces at 3 dpi (by t-test, *p* < 0.01), reaching a 0.6 log reduction. So far, there have been no reports of MNoV detection in stool of the STAT1^−/−^ mice in in vivo antiviral studies. The 0.6 log reduction of virus titers in this regard appears to be critical to improved survival rates, compared to the control mice.

The global algae production had doubled from 14.7 million tons in 2005 to 30.4 million tons in 2015. There are at least 221 species of algae of commercial value. Brown algae, LJ and UP, are intensively cultivated species for food [48] (FAO, 2018). In 2014, LJ and UP accounted for 29% and 9% of the global algae aquaculture production, respectively [49]. These two algae are important food resources, which are the most common materials consumed in raw, dried or boiled in soups and stews. They are considered to be good nutritional sources such as fiber, vitamins and minerals [48]. Fucoidans are considered to be a health-promoting compound of brown algae and have been demonstrated to protect against lipid oxidation, blood coagulation and cancer [27,28,31]. The bioactivities of fucoidans are known to be highly dependent on the composition and structure of components [50,51]. In this study, the LJ fucoidan is mostly composed of fucose, with a significant amount of sulfate and small amounts of other monosaccharides, suggesting that the LJ fucoidan is a fucose-containing sulfated polysaccharide, similar to the fucoidan of *Saccharina japonica* [52]. While the antiviral activity of fucoidans has rarely been reported, a fucoidan fraction from *Sargassum swartzii* exhibited anti-HIV-1 activity [29] and a fucoidan of *Kjellmaniella crassifolia* showed anti-influenza activity [30]. Although the LJ fucoidan showed no antibacterial activity, its depolymerized form showed significant antibacterial activity against *E. coli* and *Staphylococcus aureus* [26].

In conclusion, the LJ fucoidan showed marked inhibitory effects against NoV in cell-based plaque assays and against binding of the HuNoV GII.4 and GII.17 P domains to saliva effectively at 1000 µg/mL. Importantly, the LJ fucoidan-treated mice exhibited a significant reduction of virus titers in the feces of the STAT1^−/−^ mice and improved survival rates.

## Figures and Tables

**Table 1:** Inhibitory effects of the fucoidans from *Laminaria japonica* (LJ), *Undaria pinnatifida* (UP) and *Undaria pinnatifida* sporophyll (UPS) against murine norovirus (MNoV) and feline calicivirus (FCV).

Sample	Conc. (µg/mL)	MNoV		FCV	
		Titer (log PFU/mL)	Log Reduction	Titer (log PFU/mL)	Log Reduction
PBS		6.18 ± 0.03 ^a^	-	6.66 ± 0.03 ^a^	-
Commercial	10	6.10 ± 0.04 ^a^	0.08	6.48 ± 0.09 ^b^	0.18
	100	5.79 ± 0.09 ^b^	0.39	6.27 ± 0.02 ^c^	0.39
	1000	5.11 ± 0.08 ^c^	1.07	5.72 ± 0.03 ^d^	0.94
LJ	10	6.07 ± 0.03 ^a^	0.11	6.46 ± 0.02 ^b^	0.20
	100	5.86 ± 0.05 ^b^	0.32	6.14 ± 0.06 ^c^	0.52
	1000	4.80 ± 0.10 ^c^	1.38	5.48 ± 0.07 ^d^	1.18
UP	10	6.01 ± 0.10 ^a^	0.17	6.54 ± 0.02 ^ab^	0.12
	100	5.95 ± 0.07 ^b^	0.23	6.45 ± 0.06 ^b^	0.21
	1000	5.50 ± 0.04 ^c^	0.66	5.90 ± 0.03 ^c^	0.76
UPS	10	5.89 ± 0.12 ^bc^	0.29	6.38 ± 0.08 ^bc^	0.28
	100	5.83 ± 0.04 ^c^	0.35	6.27 ± 0.01 ^c^	0.39
	1000	5.08 ± 0.05 ^d^	1.10	5.32 ± 0.10 ^d^	1.34

Phosphate buffered saline (PBS). Fucoidans were incubated with MNoV or FCV for 3 h at room temperature and then inoculated onto the cells. The commercial fucoidan (Sigma-Aldrich, ≥95% purity) and PBS were used as positive and untreated controls, respectively. Different letters indicate significant differences between each fucoidan and PBS (*p* < 0.05).

**Table 2:** Chemical compositions of the fucoidan from *Laminaria japonica*.

	Sulfate (%)	Monosaccharides (%)					
		Fucose	Galactose	Glucose	Mannose	Rhamnose	Xylose
*Laminaria japonica*	25.8	23.6	2.8	0.3	1.5	0	0.3

## Supplementary Materials

The following are available online at https://www.mdpi.com/1999-4915/12/9/997/s1, Figure S1: Preparation of the HuNoV GII.4 and GII.17 P domains. The recombinant P domains were purified by Ni-NTA affinity and size exclusion chromatography and analysed by SDS-PAGE (inset), Figure S2: Binding of the HuNoV P domains to saliva. Binding of the HuNoV GII.4 (**A**) and GII.17 (**B**) P domains to A- or O-type saliva was determined using ELISA. All experiments were performed in triplicate. Standard deviation is shown with black bars. The P domains were shown to bind to A- and O-type saliva in a dose-dependent manner, Figure S3: Cytotoxicity of the fucoidans of *Laminaria japonica* (LJ), *Undaria pinnatifida* (UP)*,* and *Undaria pinnatifida* sporophyll (UPS). Cytotoxicity was measured by MTT assay. (**A**) RAW or (**B**) CRFK cells were treated with the fucoidans of LJ, UP, and UPS for 24 h, respectively. A commercial fucoidan (≥95% purity) was used as a positive control. The percentage of cell viability was calculated as follows: % cell viability = (Abs_treatment_/Abs_control_) × 100. Significance level was indicated by * *p* < 0.05. There was no significant difference in the cell viability of the fucoidan from LJ, UP, and UPS compared to that of RAW or CRFK cell alone.
