# Supplementary material for: Inhibitory Effects of Laminaria japonica Fucoidans Against Noroviruses
Source: Viruses. 2020 Sep 7;12(9):997. doi: 10.3390/v12090997 (PMC7552056; doi:10.3390/v12090997)
Supplement: Supplementary file 1 [file viruses-12-00997-s001.pdf]

viruses

Article

Supplementary Materials: Inhibitory effects of *Laminaria japonica* fucoidans against noroviruses

Hyojin Kim, Chae Yeon Lim, Dan Bi Lee, Jong Hyeon Seok, Kyung Hyun Kim and Mi Sook Chung

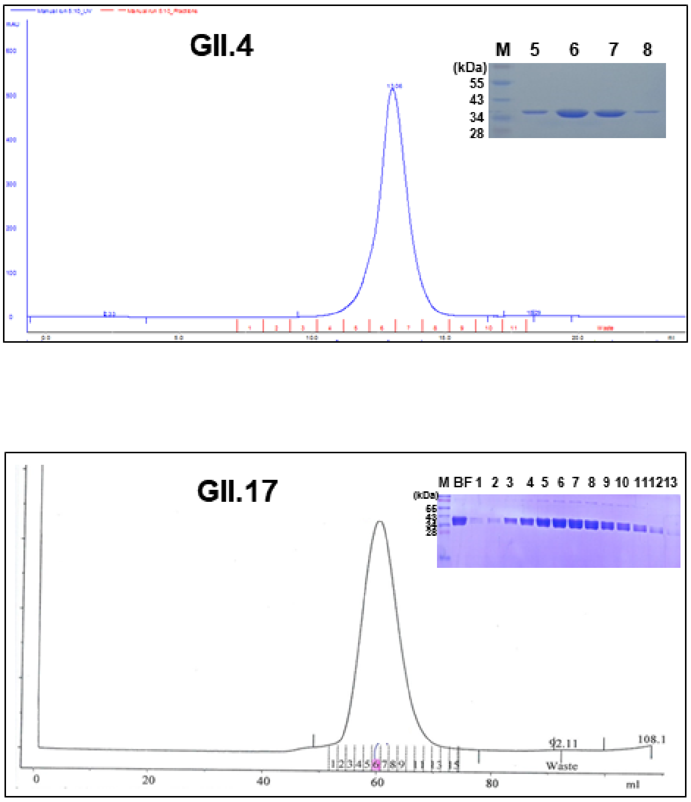

Figure S1. Preparation of the HuNoV GII.4 and GII.17 P domains. The recombinant P domains were purified by Ni-NTA affinity and size exclusion chromatography and analysed by SDS-PAGE (inset).

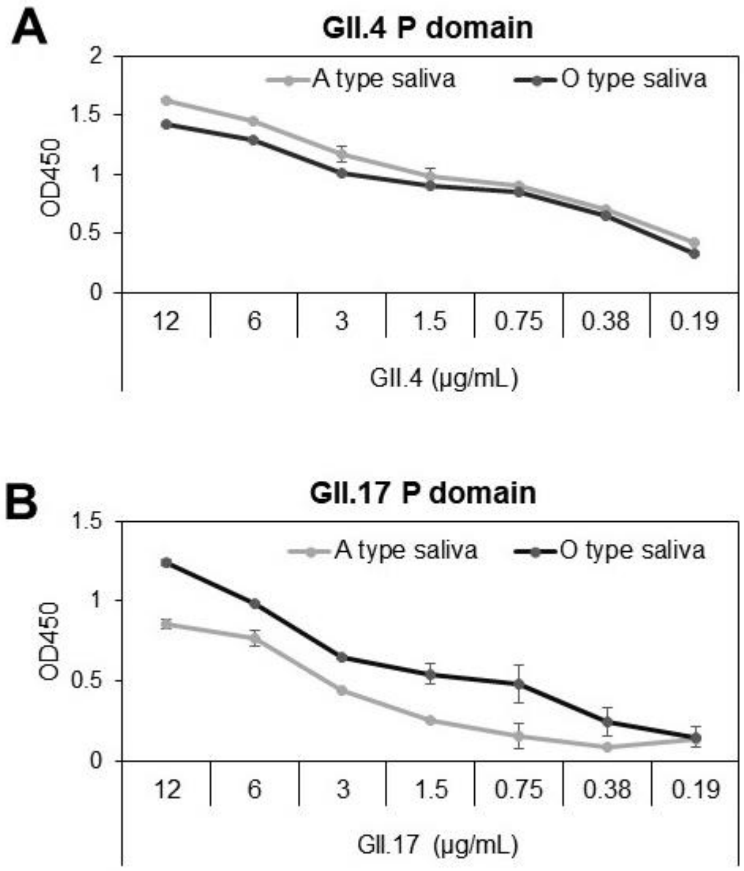

Figure S2. Binding of the HuNoV P domains to saliva. Binding of the HuNoV GII.4 (**A**) and GII.17 (**B**) P domains to A- or O-type saliva was determined using ELISA. All experiments were performed in triplicate. Standard deviation is shown with black bars. The P domains were shown to bind to A- and O-type saliva in a dose-dependent manner.

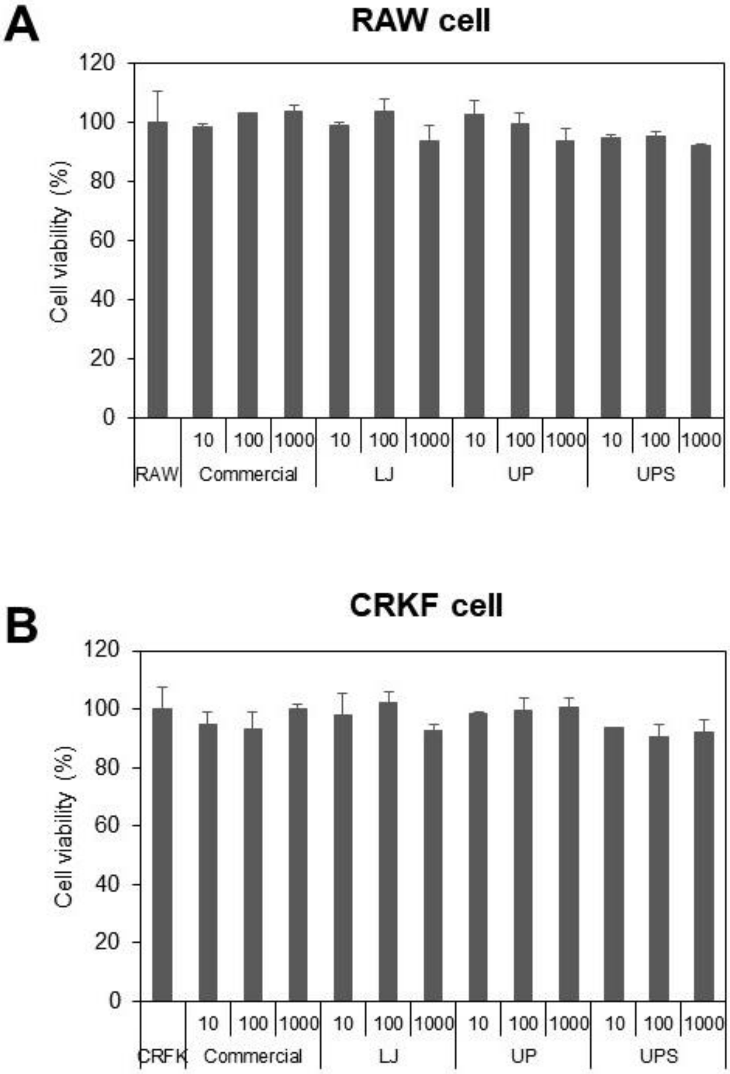

Figure S3. Cytotoxicity of the fucoidans of *Laminaria japonica* (LJ), *Undaria pinnatifida* (UP), and *Undaria pinnatifida* sporophyll (UPS). Cytotoxicity was measured by MTT assay. (**A**) RAW or (**B**) CRFK cells were treated with the fucoidans of LJ, UP, and UPS for 24 h, respectively. A commercial fucoidan (≥95% purity) was used as a positive control. The percentage of cell viability was calculated as follows: % cell viability = ($Abs_{treatment}$ /$Abs_{control}$) × 100. Significance level was indicated by $^{*}p < 0.05$. There was no significant difference in the cell viability of the fucoidan from LJ, UP, and UPS compared to that of RAW or CRFK cell alone.

© 2020 by the authors. Submitted for possible open access publication under the terms and conditions of the Creative Commons Attribution (CC BY) license (http://creativecommons.org/licenses/by/4.0/).
